# Bis(butan-1-aminium) naphthalene-1,5-disulfonate

**DOI:** 10.1107/S1600536812018880

**Published:** 2012-05-02

**Authors:** Yu Jin

**Affiliations:** a Ordered Matter Science Research Center, Southeast University, Nanjing 211189, People’s Republic of China

## Abstract

In the title compound, 2C_4_H_12_N^+^·C_10_H_6_O_6_S_2_^2−^, the anion lies on an inversion center, so the asymmetric unit contains half an anion and one cation which are linked by a strong N—H⋯O hydrogen bond. The crystal structure comprises discrete ions, which are linked into centrosymmetric *R*_4_^4^(12) loops by N—H⋯O inter­actions.

## Related literature

For related structures, see: Jin (2011*a* ▶,*b* ▶, 2012 ▶). For hydrogen-bond motifs, see: Bernstein *et al.* (1995 ▶).
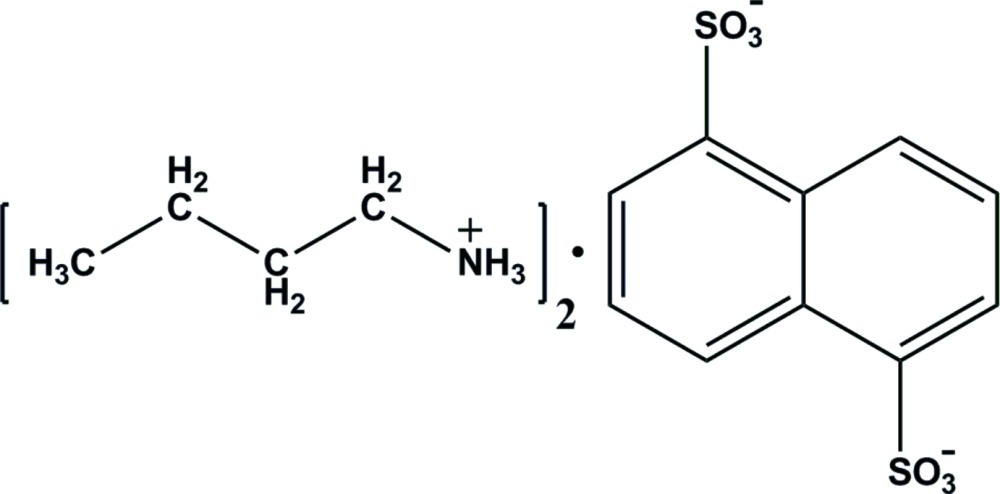


## Experimental

### 

#### Crystal data

2C_4_H_12_N^+^·C_10_H_6_O_6_S_2_^2−^
*M* *_r_* = 434.56
Monoclinic, 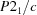

*a* = 8.1532 (16) Å
*b* = 9.2582 (19) Å
*c* = 14.108 (5) Å
β = 108.02 (3)°
*V* = 1012.7 (5) Å^3^
*Z* = 2
Mo *K*α radiation
μ = 0.30 mm^−1^
*T* = 293 K
0.3 × 0.3 × 0.2 mm

#### Data collection

Rigaku Mercury CCD diffractometer
Absorption correction: multi-scan (*CrystalClear*; Rigaku, 2005 ▶) *T*_min_ = 0.489, *T*_max_ = 1.000
10171 measured reflections
2316 independent reflections
2039 reflections with *I* > 2σ(*I*)
*R*_int_ = 0.034

#### Refinement

*R*[*F* ^2^ > 2σ(*F* ^2^)] = 0.038
*wR*(*F* ^2^) = 0.094
*S* = 1.14
2316 reflections
128 parameters
H-atom parameters constrained
Δρ_max_ = 0.24 e Å^−3^
Δρ_min_ = −0.43 e Å^−3^

### 

Data collection: *CrystalClear* (Rigaku, 2005 ▶); cell refinement: *CrystalClear*; data reduction: *CrystalClear*; program(s) used to solve structure: *SHELXS97* (Sheldrick, 2008 ▶); program(s) used to refine structure: *SHELXL97* (Sheldrick, 2008 ▶); molecular graphics: *SHELXTL* (Sheldrick, 2008 ▶); software used to prepare material for publication: *SHELXL97*.

## Supplementary Material

Crystal structure: contains datablock(s) I, global. DOI: 10.1107/S1600536812018880/bx2406sup1.cif

Structure factors: contains datablock(s) I. DOI: 10.1107/S1600536812018880/bx2406Isup2.hkl

Supplementary material file. DOI: 10.1107/S1600536812018880/bx2406Isup3.cml

Additional supplementary materials:  crystallographic information; 3D view; checkCIF report

## Figures and Tables

**Table 1:** Hydrogen-bond geometry (Å, °)

*D*—H⋯*A*	*D*—H	H⋯*A*	*D*⋯*A*	*D*—H⋯*A*
N1—H1*A*⋯O1	0.89	1.95	2.840 (2)	177
N1—H1*C*⋯O2^i^	0.89	1.97	2.857 (2)	177
N1—H1*B*⋯O3^ii^	0.89	2.05	2.911 (2)	162

Symmetry codes: (i) 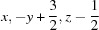; (ii) 
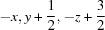
.

**Fig. 1.**
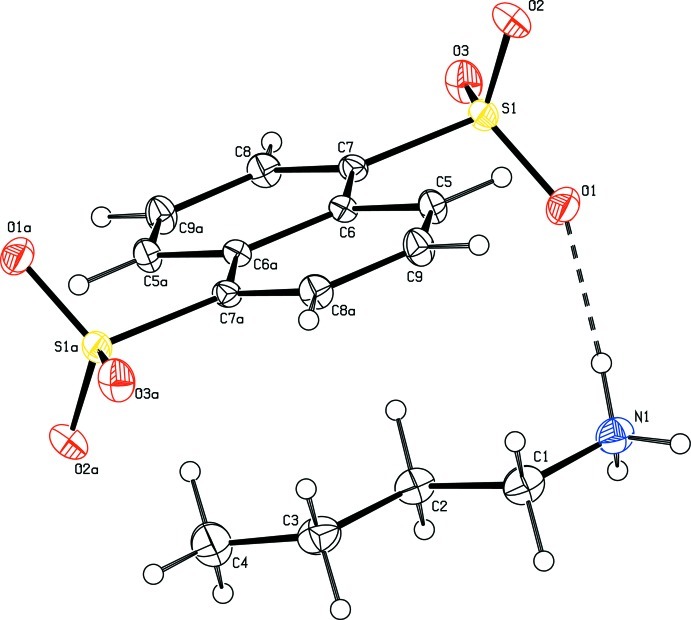
A view of the title compound showing the labelling of the non-H atoms. Displacement ellipsoids drawn at the 30% probability level. [Symmetry code: (a) 1-x, 2-y, 2-z]

**Fig. 2.**
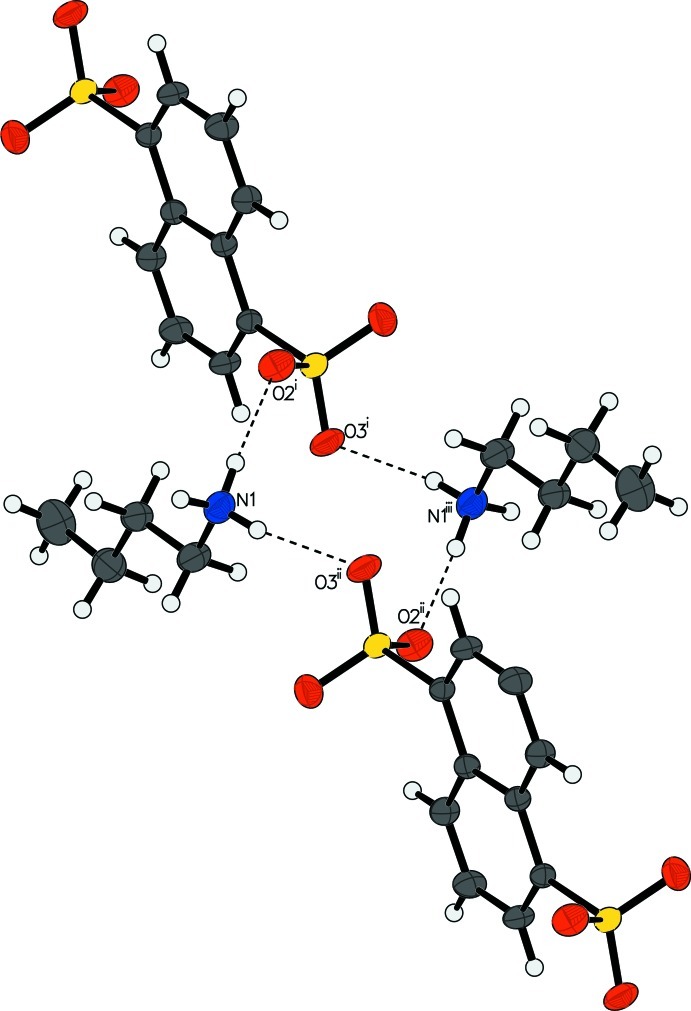
Part of the crystal structure of (I) , showing discrete ions which are linked into centrosymmetric R44(12) dimers.[Symmetry codes : (i) x, 3/2-y, -1/2+z; (ii) -x, 1/2+y, 3/2-z; (iii) -x,2-y,1-z]

## supplementary crystallographic information

### Comment

As an extension of the research about naphthalene-1,5-disulfonate salts (Jin, 2011*a*; Jin, 2011*b*; Jin, 2012), I report here the synthesis and the crystal structure of the title complex, 2C_4_H_12_N^+^C_10_H_6_S_2_O_6_^2-^. In the title compound 2 C_4_H_12_N^+ ^. C_10_H_6_S_2_O_6_^2^ , the anion lies on inversion center, so the asymmetric unit contains one-half anion and one cation which are linked by one strong N—H···O hydrogen bond interaction, Fig 1. The crystal structure comprises discrete ions which are linked into centrosymmetric *R_4_*^4^(12) dimers by simple N—H···O interactions, (Bernstein, *et al.*, 1995), Fig 2, Table1.

### Experimental

2C_4_H_12_N^+^&bull;C_10_H_6_S_2_O_6_^2-^ was synthetized from a mixture of CH_3_(CH_2_)_3_NH_2_ (146.28 mg, 2.00 mmol), C_10_H_8_O_6_S_2_ (288.28 mg, 1.00 mmol), and distilled water (10 ml), which was stirred a few minutes at room temperature, giving a clear transparent solution. After evaporation for a few days, block colorless crystals suitable for X-ray diffraction were obtained in about 88% yield and filtered and washed with distilled water.

### Refinement

H atoms bound to carbon and nitrogen were placed at idealized positions [C—H =0.93 to 0.97 Å and N—H = 0.89 Å] and allowed to ride on their parent atoms with *U*_iso_ fixed at 1.2 *U*_eq_(C,*N*).

### Crystal data

**Table:**

2C_4_H_12_N^+^·C_10_H_6_O_6_S_2_^2^^−^	*F*(000) = 464
*M**_r_* = 434.56	*D*_x_ = 1.425 Mg m^−^^3^
Monoclinic, *P*2_1_/*c*	Mo *K*α radiation, λ = 0.71073 Å
Hall symbol: -P 2ybc	Cell parameters from 3450 reflections
*a* = 8.1532 (16) Å	θ = 6.2–55.3°
*b* = 9.2582 (19) Å	µ = 0.30 mm^−^^1^
*c* = 14.108 (5) Å	*T* = 293 K
β = 108.02 (3)°	Block, colourless
*V* = 1012.7 (5) Å^3^	0.3 × 0.3 × 0.2 mm
*Z* = 2	

### Data collection

**Table:**

Rigaku Mercury CCD diffractometer	2316 independent reflections
Radiation source: fine-focus sealed tube	2039 reflections with *I* > 2σ(*I*)
Graphite monochromator	*R*_int_ = 0.034
ω scans	θ_max_ = 27.5°, θ_min_ = 3.0°
Absorption correction: multi-scan (*CrystalClear*; Rigaku, 2005)	*h* = −10→10
*T*_min_ = 0.489, *T*_max_ = 1.000	*k* = −12→12
10171 measured reflections	*l* = −18→18

### Refinement

**Table:**

Refinement on *F*^2^	Primary atom site location: structure-invariant direct methods
Least-squares matrix: full	Secondary atom site location: difference Fourier map
*R*[*F*^2^ > 2σ(*F*^2^)] = 0.038	Hydrogen site location: inferred from neighbouring sites
*wR*(*F*^2^) = 0.094	H-atom parameters constrained
*S* = 1.14	*w* = 1/[σ^2^(*F*_o_^2^) + (0.0379*P*)^2^ + 0.4216*P*] where *P* = (*F*_o_^2^ + 2*F*_c_^2^)/3
2316 reflections	(Δ/σ)_max_ = 0.001
128 parameters	Δρ_max_ = 0.24 e Å^−^^3^
0 restraints	Δρ_min_ = −0.43 e Å^−^^3^

### Special details

**Table:**

Geometry. All e.s.d.'s (except the e.s.d. in the dihedral angle between two l.s. planes) are estimated using the full covariance matrix. The cell e.s.d.'s are taken into account individually in the estimation of e.s.d.'s in distances, angles and torsion angles; correlations between e.s.d.'s in cell parameters are only used when they are defined by crystal symmetry. An approximate (isotropic) treatment of cell e.s.d.'s is used for estimating e.s.d.'s involving l.s. planes.
Refinement. Refinement of *F*^2^ against ALL reflections. The weighted *R*-factor *wR* and goodness of fit *S* are based on *F*^2^, conventional *R*-factors *R* are based on *F*, with *F* set to zero for negative *F*^2^. The threshold expression of *F*^2^ > σ(*F*^2^) is used only for calculating *R*-factors(gt) *etc*. and is not relevant to the choice of reflections for refinement. *R*-factors based on *F*^2^ are statistically about twice as large as those based on *F*, and *R*- factors based on ALL data will be even larger.

### Fractional atomic coordinates and isotropic or equivalent isotropic displacement parameters (Å^2^)

**Table:**

	*x*	*y*	*z*	*U*_iso_*/*U*_eq_	
C1	0.2319 (2)	1.0297 (2)	0.69402 (14)	0.0364 (4)	
H1D	0.2261	1.0939	0.7473	0.044*	
H1E	0.2235	1.0880	0.6356	0.044*	
C2	0.4015 (2)	0.95077 (19)	0.72490 (13)	0.0332 (4)	
H2A	0.4062	0.8866	0.6714	0.040*	
H2B	0.4085	0.8919	0.7829	0.040*	
C3	0.5553 (3)	1.0519 (2)	0.74938 (15)	0.0405 (5)	
H3A	0.5439	1.1157	0.6932	0.049*	
H3B	0.5553	1.1112	0.8061	0.049*	
C4	0.7256 (3)	0.9722 (3)	0.77313 (17)	0.0524 (6)	
H4A	0.7286	0.9168	0.7161	0.079*	
H4B	0.7376	0.9087	0.8286	0.079*	
H4C	0.8186	1.0406	0.7896	0.079*	
C5	0.2728 (2)	1.06642 (17)	0.97032 (12)	0.0253 (3)	
H5	0.1617	1.0294	0.9555	0.030*	
C6	0.41452 (18)	0.97062 (16)	0.98793 (11)	0.0198 (3)	
C7	0.39614 (19)	0.81664 (16)	0.98343 (11)	0.0204 (3)	
C8	0.5368 (2)	0.72878 (17)	1.00271 (12)	0.0259 (3)	
H8	0.5228	0.6290	1.0009	0.031*	
C9	0.2973 (2)	1.21166 (18)	0.97477 (13)	0.0286 (4)	
H9	0.2025	1.2727	0.9627	0.034*	
N1	0.0850 (2)	0.92591 (16)	0.67115 (11)	0.0338 (3)	
H1A	0.0840	0.8815	0.7269	0.041*	
H1B	−0.0136	0.9735	0.6455	0.041*	
H1C	0.0971	0.8608	0.6274	0.041*	
O1	0.09524 (16)	0.78595 (15)	0.85199 (9)	0.0380 (3)	
O2	0.11042 (16)	0.77995 (14)	1.02545 (9)	0.0343 (3)	
O3	0.21663 (16)	0.57911 (13)	0.95327 (10)	0.0353 (3)	
S1	0.18812 (5)	0.73440 (4)	0.95082 (3)	0.02387 (13)	

### Atomic displacement parameters (Å^2^)

**Table:**

	*U*^11^	*U*^22^	*U*^33^	*U*^12^	*U*^13^	*U*^23^
C1	0.0436 (11)	0.0273 (9)	0.0380 (10)	0.0057 (8)	0.0123 (8)	0.0024 (7)
C2	0.0409 (10)	0.0277 (9)	0.0313 (9)	0.0033 (7)	0.0118 (8)	−0.0002 (7)
C3	0.0491 (12)	0.0362 (10)	0.0351 (10)	−0.0046 (9)	0.0113 (9)	0.0010 (8)
C4	0.0407 (12)	0.0699 (16)	0.0470 (12)	−0.0053 (11)	0.0139 (10)	−0.0027 (11)
C5	0.0186 (7)	0.0255 (8)	0.0320 (8)	−0.0005 (6)	0.0078 (6)	0.0015 (6)
C6	0.0197 (7)	0.0203 (7)	0.0206 (7)	−0.0011 (6)	0.0081 (6)	0.0008 (5)
C7	0.0212 (7)	0.0202 (7)	0.0204 (7)	−0.0037 (6)	0.0073 (6)	0.0008 (6)
C8	0.0282 (8)	0.0166 (7)	0.0333 (8)	−0.0006 (6)	0.0101 (7)	0.0011 (6)
C9	0.0225 (8)	0.0235 (8)	0.0400 (9)	0.0052 (6)	0.0100 (7)	0.0025 (7)
N1	0.0363 (8)	0.0338 (8)	0.0320 (8)	0.0092 (6)	0.0116 (6)	0.0033 (6)
O1	0.0318 (7)	0.0443 (8)	0.0306 (7)	−0.0089 (6)	−0.0010 (5)	0.0079 (5)
O2	0.0315 (6)	0.0355 (7)	0.0417 (7)	−0.0059 (5)	0.0198 (6)	−0.0001 (5)
O3	0.0332 (7)	0.0219 (6)	0.0481 (8)	−0.0085 (5)	0.0084 (6)	−0.0019 (5)
S1	0.0220 (2)	0.0218 (2)	0.0267 (2)	−0.00585 (14)	0.00604 (15)	0.00118 (15)

### Geometric parameters (Å, º)

**Table:**

C1—N1	1.491 (2)	C5—H5	0.9300
C1—C2	1.505 (3)	C6—C7	1.433 (2)
C1—H1D	0.9700	C6—C6^i^	1.436 (3)
C1—H1E	0.9700	C7—C8	1.363 (2)
C2—C3	1.517 (3)	C7—S1	1.7847 (15)
C2—H2A	0.9700	C8—C9^i^	1.403 (2)
C2—H2B	0.9700	C8—H8	0.9300
C3—C4	1.516 (3)	C9—C8^i^	1.403 (2)
C3—H3A	0.9700	C9—H9	0.9300
C3—H3B	0.9700	N1—H1A	0.8900
C4—H4A	0.9600	N1—H1B	0.8900
C4—H4B	0.9600	N1—H1C	0.8900
C4—H4C	0.9600	O1—S1	1.4464 (14)
C5—C9	1.358 (2)	O2—S1	1.4493 (13)
C5—C6	1.416 (2)	O3—S1	1.4550 (13)
			
N1—C1—C2	110.73 (15)	C6—C5—H5	119.6
N1—C1—H1D	109.5	C5—C6—C7	123.16 (14)
C2—C1—H1D	109.5	C5—C6—C6^i^	118.93 (17)
N1—C1—H1E	109.5	C7—C6—C6^i^	117.91 (17)
C2—C1—H1E	109.5	C8—C7—C6	120.99 (14)
H1D—C1—H1E	108.1	C8—C7—S1	118.08 (12)
C1—C2—C3	112.76 (16)	C6—C7—S1	120.93 (11)
C1—C2—H2A	109.0	C7—C8—C9^i^	120.22 (15)
C3—C2—H2A	109.0	C7—C8—H8	119.9
C1—C2—H2B	109.0	C9^i^—C8—H8	119.9
C3—C2—H2B	109.0	C5—C9—C8^i^	121.16 (15)
H2A—C2—H2B	107.8	C5—C9—H9	119.4
C4—C3—C2	112.72 (18)	C8^i^—C9—H9	119.4
C4—C3—H3A	109.0	C1—N1—H1A	109.5
C2—C3—H3A	109.0	C1—N1—H1B	109.5
C4—C3—H3B	109.0	H1A—N1—H1B	109.5
C2—C3—H3B	109.0	C1—N1—H1C	109.5
H3A—C3—H3B	107.8	H1A—N1—H1C	109.5
C3—C4—H4A	109.5	H1B—N1—H1C	109.5
C3—C4—H4B	109.5	O1—S1—O2	112.83 (9)
H4A—C4—H4B	109.5	O1—S1—O3	112.40 (8)
C3—C4—H4C	109.5	O2—S1—O3	111.89 (8)
H4A—C4—H4C	109.5	O1—S1—C7	106.27 (8)
H4B—C4—H4C	109.5	O2—S1—C7	106.42 (8)
C9—C5—C6	120.77 (15)	O3—S1—C7	106.46 (7)
C9—C5—H5	119.6		
			
N1—C1—C2—C3	−179.79 (15)	S1—C7—C8—C9^i^	177.76 (13)
C1—C2—C3—C4	−175.79 (16)	C6—C5—C9—C8^i^	−0.3 (3)
C9—C5—C6—C7	−179.48 (15)	C8—C7—S1—O1	−119.32 (13)
C9—C5—C6—C6^i^	0.9 (3)	C6—C7—S1—O1	59.88 (14)
C5—C6—C7—C8	−178.83 (15)	C8—C7—S1—O2	120.19 (13)
C6^i^—C6—C7—C8	0.8 (3)	C6—C7—S1—O2	−60.61 (14)
C5—C6—C7—S1	2.0 (2)	C8—C7—S1—O3	0.70 (15)
C6^i^—C6—C7—S1	−178.37 (13)	C6—C7—S1—O3	179.90 (12)
C6—C7—C8—C9^i^	−1.4 (2)		

Symmetry code: (i) −*x*+1, −*y*+2, −*z*+2.

### Hydrogen-bond geometry (Å, º)

**Table:**

*D*—H···*A*	*D*—H	H···*A*	*D*···*A*	*D*—H···*A*
N1—H1*A*···O1	0.89	1.95	2.840 (2)	177
N1—H1*C*···O2^ii^	0.89	1.97	2.857 (2)	177
N1—H1*B*···O3^iii^	0.89	2.05	2.911 (2)	162

Symmetry codes: (ii) *x*, −*y*+3/2, *z*−1/2; (iii) −*x*, *y*+1/2, −*z*+3/2.
